# EEG Based Monitoring of General Anesthesia: Taking the Next Steps

**DOI:** 10.3389/fncom.2017.00056

**Published:** 2017-06-22

**Authors:** Matthias Kreuzer

**Affiliations:** ^1^Department of Anesthesiology, Emory University School of MedicineAtlanta, GA, United States; ^2^Research Division, Atlanta VA Medical CenterAtlanta, GA, United States

**Keywords:** EEG, anesthesia, monitoring, delirium, adverse outcomes

Electroencephalographic recordings (EEG) present an opportunity to monitor changes in human brain electrical activity during changing states of consciousness like sleep or general anesthesia. Frontal EEG recordings during surgical interventions with anesthetic-induced unconsciousness help to estimate the patients' level of (un)consciousness.

## EEG-based monitoring of the level of consciousness: commercial devices

The classical way to extract information from the recorded EEG relevant for assessment of the level of anesthesia is the application of algorithms that evaluate changes in the oscillatory behavior of the EEG that is mainly derived from frontal EEG montages placed on the patients' forehead. These calculations are most often performed in the frequency domain, i.e., after transformation of the signal, e.g., by the Fourier Transform. The most prominent commercial system, the bispectral index (BIS, Medtronic, Dublin, Ireland) evaluates changes in the log ratio of the 30 to 47 Hz and 11 to 20 Hz EEG band power (BetaRatio) as well as a ratio of the sum of bispectrum peaks in the 0.5 to 47 Hz and the 40 to 47 Hz range (SynchFastSlow) (Rampil, 1998). The bispectrum presents a higher order spectrum that evaluates the phase correlation of different frequency components and is able to identify nonlinear signal properties (Rampil, 1998). The BetaRatio subparameter outperforms SynchFastSlow and BIS in separating consciousness from unconsciousness (Schneider et al., 2004). SynchFastSlow dominates BIS calculation during surgical levels of anesthesia (Rampil, 1998). State and Response entropy (GE Healthcare, Chicago, IL) evaluate changes in the shape of the power spectrum (Viertio-Oja et al., 2004). Other devices like the CSI (Danmeter, Odense, Denmark), IoC (Morpheus Medical, Barcelona, Spain), or qCON (Quantium Medical, Mataro, Spain) use ratios of EEG band power (Jensen et al., 2006, 2014; Revuelta et al., 2008). The IoC also processes information from the EEG after transformation to a time series of symbols (Revuelta et al., 2008). The Narcotrend (Narcotrend, Hannover, Germany) utilizes information from the spectral domain as well from autoregressive modeling in the time domain (Kreuer and Wilhelm, 2006). The PSI from the SEDLine monitor (Masimo, Irvine, CA) processes spectral power from different frequency bands as well as interhemispheric power gradients and synchrony (Prichep et al., 2004). The Brain Anesthesia Response (BAR) monitor (Cortical Dynamics Ltd., North Perth, Australia) takes a different approach. It generates its index by modeling EEG dynamics (Liley et al., 2010b). These devices have in common, that combining the subparameters is performed by a proprietary algorithm, and hence the contribution of each parameter to the index is not known. In general, these indices track the suppression of high frequency EEG activity and the activation of low frequent oscillations, as triggered by many common anesthetics (Brown et al., 2010). By using spectral power, or parameters derived from it, as key parameters, the monitoring systems may dismiss signal information content by neglecting the phase component of the signals' frequency and only exploiting information from the amplitude spectrum (Callegaro, 2012). Further, the devices susceptibility to muscle activity (Messner et al., 2003; Schuller et al., 2015), especially by including high EEG frequencies as well as the time delay, necessary for index calculation (Pilge et al., 2006; Zanner et al., 2009; Kreuzer et al., 2012) may present a limiting factor in performance to reliably track the anesthetic state.

## EEG-based consciousness monitoring in research: time-domain analytical approaches

**Figure 1:**
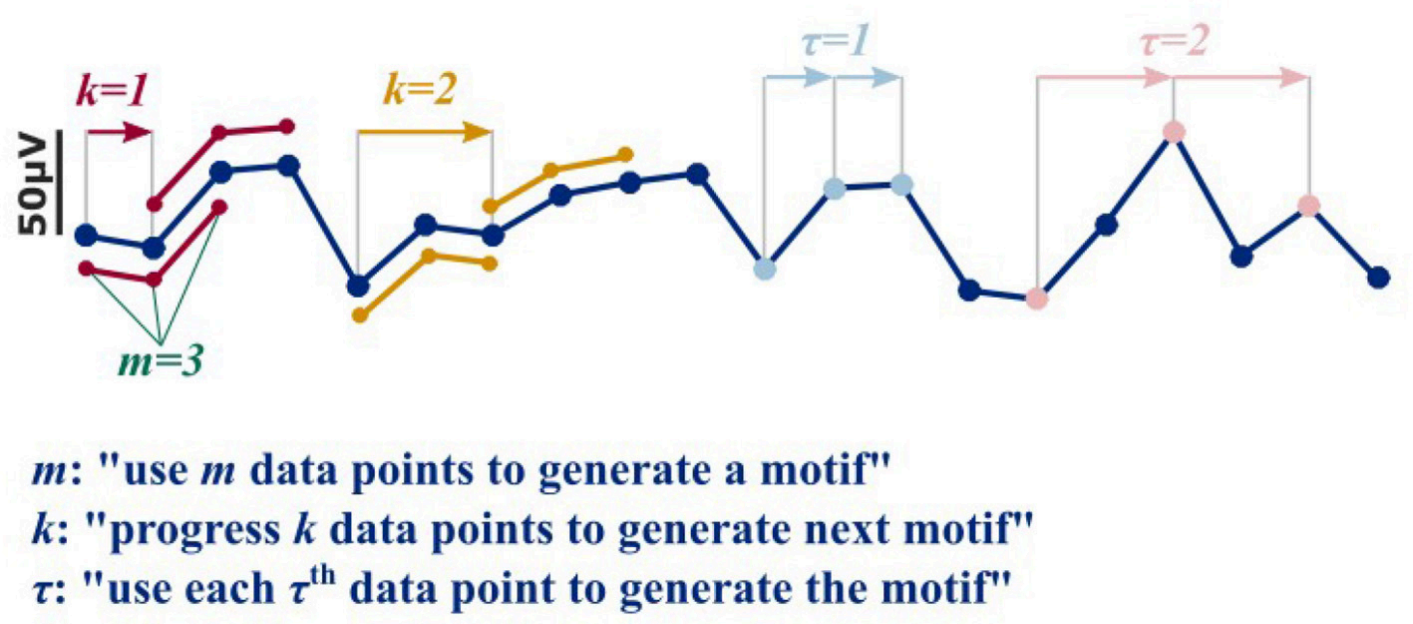
In order to generate a motif as used for the nonlinear, entropy based approaches, motive length *m*, time delay τ, and shift *k* have to be defined. The parameter *k* defines the shift. For a *k* = 1, the first motif of length *m* = 3 starts at data point *i*, and the second at *i*+*k* = 2 and so on (red). For a *k* of 2, the first motif would start at data point *i*, and the second at *i*+*k* = 3, and so on (yellow). The parameter τ defines how many data points are left out to generate the motif. E.g., for a τ = 1 and *m* = 3, the data points *i, i*+1, and *i*+2 are used to generate the motif (light blue). For a τ = 1, the data points *i, i*+2, and *i*+4 are used (pink).

More recent approaches to extract information from the EEG at different levels of anesthesia use nonlinear parameters that reflect signal information content, complexity, and/or predictability. These approaches seem capable to extract non-linear information from the signal as investigated with surrogate techniques, while linear measures like spectral entropy or the Hurst exponent did not detect these non-linearities (Jordan et al., 2009; Anier et al., 2010). In EEG and anesthesia research the most prominent players are approximate entropy (ApEn) (Pincus, 1991; Bruhn et al., 2000) and permutation entropy (PeEn) (Bandt and Pompe, 2002; Jordan et al., 2008; Olofsen et al., 2008) for single channel analysis and cross approximate entropy (Pincus Steven et al., 1996; Hudetz, 2002; Kreuzer et al., 2010), (symbolic) transfer entropy (Schreiber, 2000; Imas et al., 2005; Staniek and Lehnertz, 2008; Jordan et al., 2013), or order recurrence plots (Groth, 2006) for bivariate analysis. These measures are applied to the EEG time domain, usually after band-pass filtering of the EEG to a wide frequency range with a low pass filter set to around 25 to 30 Hz to limit EEG signal contamination by electromyographic activity (EMG). Frontal EMG activity can occur in the entire frequency range but seems to peak between 25 and 30 Hz (Goncharova et al., 2003). The mentioned entropy measures usually define consecutive amplitude values or their ranks as pieces of information, called motifs. The EEG is then represented as series of motifs. The user can define the length *m* of a motif (the number of amplitude values it is generated from), a time lag parameter τ (to consider only each τth amplitude value to define a motif of length *m*), and a shift *k* (to shift *k* amplitude values from the first amplitude value of the previous motif to start generation of the next motif of length *m* with lag τ). Figure 1 presents the impact of *k* and τ on motif generation.

The transfer entropies that quantify directed information flow include another parameter δ (Staniek and Lehnertz, 2008) to define transfer lags or transmission time of the motif of information between the two channels. The time lag parameters δ may evaluate changes in signal information roughly associated with a certain frequency range. When compared to spectral approaches and commercial monitors, the univariate measures ApEn and PeEn showed higher performance in distinguishing EEG recorded during consciousness from EEG recorded during unconsciousness and to reflect different levels of general anesthesia (Bruhn et al., 2000; Jordan et al., 2008; Liang et al., 2015). A newly proposed, multimodal index, integrates PeEn to separate consciousness from unconsciousness and ApEn to scale different levels of anesthesia (Schneider et al., 2014). The use of the bivariate transfer entropies revealed a loss of cortical feedback connectivity as a key mechanism of anesthetic-induced unconsciousness that is universal for most anesthetics (Ku et al., 2011; Jordan et al., 2013; Lee et al., 2013; Ranft et al., 2016). Interestingly the parameter settings were targeted toward the EEG beta frequency range. This frequency range may play an important role in synchronizing different cortical regions (von Stein and Sarnthein, 2000; Bassett et al., 2009; Hipp et al., 2011). So although these nonlinear parameters are applied to a wide frequency range, their intrinsic setting defines the information to be extracted from the signal.

## Different entropies evaluate different properties

**Figure 2:**
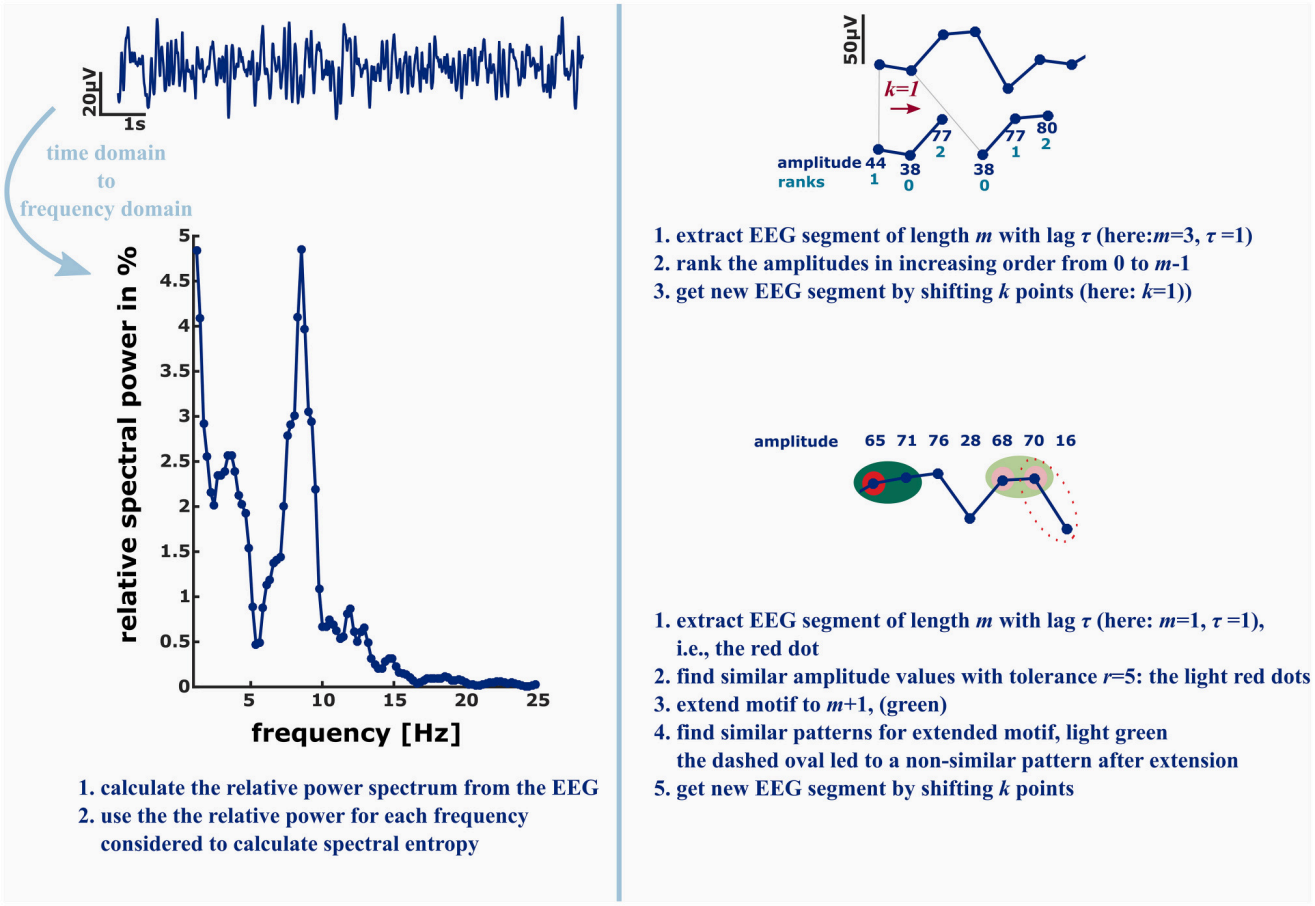
**Left:** in order to calculate the spectral entropy as for example used in the GE Entropy Module, the EEG power spectrum is calculated from the recording. The spectral entropy value reflects the shape of the power spectrum. The more uniformly distributed the power is among the frequencies, the higher is the spectral entropy value. **Right:** permutation entropy (PeEn, top) and approximate entropy (ApEn, bottom) in contrast are directly derived from the EEG time series. For the ordinal PeEn motifs of length *m* are represented as a series of ranks, with the lowest amplitude value being equal to rank 0 and the highest amplitude value being equal to rank *m*−1.

These findings are a strong claim to include nonlinear analysis techniques in commercial “depth of anesthesia” monitoring as well as to extend the EEG electrode layout to at least one electrode placed in parietal or occipital regions to be able to monitor the loss of cortical feedback activity. Further, there is something to be mentioned regarding “entropy analysis” in anesthesiology. Often, for instance at conferences there is just a discussion about “entropy” analysis without defining what method really has been used. These measures, even if they share the term “entropy” analyze different signal features. A very prominent example is the spectral entropy, a measure evaluating the change in shape of the power spectrum (Viertio-Oja et al., 2004). It evaluates the changes in the frequency domain, so it cannot be compared to analytical techniques in the time domain. Another example is the difference between ApEn and PeEn. The ordinal PeEn evaluates the probability distribution of amplitude rank patterns in the signal, while ApEn evaluates the probability of similar absolute amplitude patterns detected in the signal remain similar if they are extended by one more amplitude value. In order to define similarity of two absolute amplitude values, the algorithm contains a tolerance that acts like a low pass filter on the signal, while the formation of rank pattern in the PeEn is more like a high pass that removes slow underlying trends in amplitude from the signal. Figure 2 presents a graphical example of the described differences. As mentioned earlier, ApEn and PeEn have different strengths. ApEn seems strong in scaling different levels of unconsciousness, while PeEn presents a strong parameter to separate consciousness from unconsciousness (Schneider et al., 2014).

Hence, the EEG time series is converted to a series of rank patterns. The more uniform the probability distribution of the *m*! possible rank patterns, the higher is PeEn. ApEn evaluates the predictability of a time series by evaluating the occurrence of similar patterns of length m. Similar means that the maximum difference of the EEG amplitude values is smaller than a tolerance *r*. The concept of ApEn is to evaluate the probability, that if a similar pattern of length *m* was detected, the patterns extended to *m*+1 will be similar as well. The higher this probability, the lower ApEn will be.

## Proposed relationships between EEG frequency and communication

Although the nonlinear approaches seem to reflect the level of consciousness in a superior way, there is a strong point in favor of continuing to use spectral analyses, together with the aforementioned approaches to optimize monitoring. It is the assumption that (frontal) EEG oscillations of certain frequencies seem to correlate with interactions of the monitored cortical area with other cortical or subcortical areas. In general, the EEG mainly reflects cortical activity (Fisch and Spehlmann, 1999), but this cortical activity also carries information from subcortical regions. Frontal EEG theta power for instance seems associated with working memory (Klimesch et al., 1994; Summerfield and Mangels, 2005). The prominent alpha peak that develops in the EEG power spectrum during general anesthesia is potentially caused by synchronous activity in the thalamocortical loop (John and Prichep, 2005; Ching et al., 2010). But this thalamocortical relationship and the contribution of each region to alpha EEG is controversially discussed, as nicely reviewed by Liley and coworkers. The thalamus may not present the principal source because thalamocortical projections are sparse, the amplitude of thalamocortical excitatory postsynaptic potentials is small, the corticocortical activity is more coherent than thalamocortical activity, the isolated cortex is able to generate rhythmic oscillations, and drugs may modulate alpha oscillations in cortex and thalamus in a different way (Liley et al., 2010a). As mentioned earlier, activity in EEG beta-band may play a role in synchronizing cortical regions (von Stein et al., 1999; John and Prichep, 2005; Bassett et al., 2009; Hipp et al., 2011). Hence changes in these frequency bands' spectral power may help to understand anesthetic-induced changes in brain activity among different regions and possibly target different components of general anesthesia. These relationships between EEG frequency and the brain's communication structure may help future research to improve EEG based patient monitoring in anesthesia with a new focus on adding an “anesthesia quality” component to monitoring, i.e., to associate EEG recorded during anesthesia maintenance and emergence with adverse outcomes like pain or delirium following anesthesia.

## Correlation of intraoperative EEG markers and adverse outcomes

The current monitoring systems as well as the presented results using nonlinear approaches to EEG-based monitoring focus on a reliable separation of different hypnotic levels that range from “fully awake” to “(burst) suppression.” Hence, these monitoring systems/approaches may be able to prevent too deep levels of general anesthesia. Prevention of too deep anesthesia may help to reduce delirious outcomes (Chan et al., 2013). But there is no algorithm component that specifically deals with the detection of intraoperative EEG markers that may be associated with postoperative adverse outcomes. There seems increasing evidence that investigation of EEG alpha-band activity may present a good start to research intraoperative EEG and its association with post-anesthetic adverse outcomes, at least for the commonly used propofol and inhaled ethers. For anesthesia emergence, results suggest that returning from anesthesia-induced unconsciousness may be more complex than anesthesia induction. The patients' EEG can follow different emergence trajectories that put patients at higher or lower risk when it comes to adverse outcomes in the postoperative care unit. Patients that abruptly transition from spectral EEG patterns of unconsciousness to spectral “wake” EEG seem more vulnerable to express pain and delirium in the postoperative care unit than patients that show episodes of non-slow wave anesthesia during emergence (Chander et al., 2014; Hight et al., 2014; Garcia et al., 2016; Kreuzer et al., 2017). During anesthesia maintenance patients most often develop a so called alpha peak in frontal EEG for the most common anesthetics propofol and sevoflurane that seems to reflect reverberations in the thalamocortical loop at least in part caused by hyperpolarization of the thalamus (Akeju et al., 2014b). Evaluation of spectral alpha peak properties may help to estimate “anesthesia quality.” Strong surgical stimulation can cause a reduction of the peak (Kochs et al., 1994) and may even lead to disappearance of the peak. Because of the possible association of alpha oscillations with the thalamus, this reduction may be caused by desynchronization of thalamocortical activity that may represent arousal (McCormick and Bal, 1997).

Patients that are not expressing strong alpha power during anesthesia or react to surgical stimulation in a stronger fashion may be at higher risk of delirium in postoperative care unit (PACU-D, unpublished data). Although PACU-D is a transient phenomenon current results highlight the association with postoperative long-term complications (Card et al., 2015; Garcia et al., 2016). Hence, avoiding or detecting PACU-D as early as possible may help to decrease the risk of developing long-term adverse outcomes. Information from the EEG alpha range may help to identify this subset of patients at risk. The correlation of lower alpha power and PACU-D may reflect a patient population with a “frailer” brain that is not able to maintain a state of stable thalamocortical synchronization. So it would definitely make sense to additionally monitor the patients' EEG reaction following surgical stimuli, adding a nociception component to EEG-based monitoring.

## There is (almost) no EEG based (combined hypnosis and) analgesia/nociception monitoring

Hagihira et al. showed that bicoherence peaks around 10 Hz and around 20 Hz, that are typical for gas anesthesia, decrease with noxious stimulation if no opioid is given (Hagihira et al., 2004). But these observations have not been used for current monitors of nociception. These devices use processed EEG like the BIS as subparameters (Ellerkmann et al., 2013; Castro et al., 2016), a wide range of spectral band power (Jensen et al., 2014), or non EEG information from heart rate variability (Ledowski et al., 2013), modeled drug and opioid concentrations (Luginbühl et al., 2010), or the polysynaptic spinal withdrawal reflex (Von Dincklage et al., 2012). One exception is the BAR. It uses two measures, cortical state and cortical input that are designed to reflect the hypnotic and analgesic component of anesthesia (Liley et al., 2010b). The Cortical Dynamics website claims that BAR detect the effects of a range of analgesic agents and hence lead the way toward a combined EEG-based analgesia and anesthesia monitoring.

Besides the susceptibility of the intraoperative EEG alpha peak to stimulation, age also influences EEG power. There is a negative absolute alpha power and total frontal EEG power to age relationship (Klimesch, 1997; Purdon et al., 2015) and age presents a risk factor for the development of delirious outcomes (Deiner and Silverstein, 2009) after general anesthesia. Cortical thinning seems to occur with age (Fjell et al., 2009). Consequently the number of (pyramidal) neurons and the number of synapses decreases as well (Teplan, 2002). The decrease in volume and neurons may present a reason for the observed reduction on total EEG power. As a consequence, the brain's communication may become more fragile and less robust to influences like surgical stimuli. All these associations indicate the usefulness to pay attention to what is happening to EEG alpha oscillations during general anesthesia maintenance and emergence. Previous research and commercial applications for monitoring anesthesia have not specifically focused on this EEG frequency range, as mentioned earlier. The addition of information extracted from the EEG alpha range may help to include a factor predictive for adverse outcomes to “depth of anesthesia” monitoring. All the findings regarding adverse outcomes base on frequency domain analyses. Additional information from nonlinear analytical approaches in the time domain may help to optimize and improve intraoperative monitoring to identify patients at risk for adverse outcomes in the future. While this article mainly deals with the EEG alpha range, probably numerous other markers in other EEG frequencies from frontal and non-frontal electrode locations exist that may help to optimize monitoring. Further the described findings are probably not valid for certain drugs like (S-)ketamine or dexmeditomitine that affect EEG activity in completely different ways than sevoflurane or propofol (Maksimow et al., 2006; Akeju et al., 2014a).

I think that around 20 years after the introduction of EEG based anesthesia monitoring to the operating room and ongoing optimization of analytical algorithms, the inclusion to consider the well-being of the patient in the postoperative period seems the logical next step. Recent and future findings from the correlation of intraoperative EEG (alpha) activity may help to introduce a new generation of anesthesia monitoring. It may present the transition from EEG-based “depth of anesthesia” to “quality of anesthesia” monitoring.

## Author contributions

The author confirms being the sole contributor of this work and approved it for publication.

### Conflict of interest statement

The author declares that the research was conducted in the absence of any commercial or financial relationships that could be construed as a potential conflict of interest.
